# Statewide Dissemination of an Evidenced-Based Email Walking Program Delivered Through Cooperative Extension

**DOI:** 10.3389/fpubh.2020.00078

**Published:** 2020-03-11

**Authors:** Elizabeth A. Richards, Stephanie Woodcox, Anna Forster

**Affiliations:** ^1^School of Nursing, Purdue University, West Lafayette, IN, United States; ^2^Cooperative Extension, Purdue University, West Lafayette, IN, United States

**Keywords:** RE-AIM, physical activity, implementation, social cognitive theory, walking, cooperative extension

## Abstract

Using the RE-AIM framework, this study evaluates the statewide dissemination of an evidenced-based, email-delivered physical activity intervention implemented through Cooperative Extension. The *Get WalkIN'* program is comprised of 16 email messages sent over 12 weeks. Email messages target social cognitive theory constructs of self-efficacy, goal-setting, self-monitoring, and social support. Program reach, effectiveness, adoption, implementation, and maintenance were assessed with quantitative measures in a pre-post design. Findings indicate that within the Extension system, program adoption was high and program maintenance was moderate. Program effectiveness was demonstrated with an increase of 77.1 ± 49.9 min in weekly walking post-program. This increase in walking was maintained 3 months post-program. Capturing data on the implementation process was challenging. Overall, the results indicate success in program adoption and maintenance with further efforts needed to improve follow-up data collection from participants.

## Introduction

Despite the strong evidence of the physical, mental, and social health benefits of meeting national physical activity guidelines, a majority of U.S. adults remain inactive (1). United States' national physical activity guidelines recommend all adults achieve at least 150 min of moderate-intensity or 75 min of vigorous-intensity physical activity each week (2). Unfortunately, in 2018 only about 20% of U.S. adults met these national recommendations (3).

Walking is particularly suitable as a population-wide physical activity promotion approach because it is inexpensive and does not require the use of specialized equipment, aside from adequate shoes (1). Also, starting a walking routine does not require specific training, has a low rate of injury, and can be completed year-round across settings (1). Furthermore, walking behavior can be easily tracked and measured so that goals or targets can be clearly defined and monitored.

On a population level, changing health behaviors such as physical activity should focus on strategies that are effective, have a wide reach, and foster behavior change maintenance. Community-based physical activity interventions using informational approaches have demonstrated some success in increasing physical activity behavior across populations (4, 5). These approaches include providing information to motivate and enable people to increase and maintain physical activity behaviors (4, 6). Community programs that deliver informational approaches through diverse media including email, newspaper, and radio have demonstrated effectiveness in increasing physical activity among adults (4, 5, 7). However, once physical activity interventions have demonstrated effectiveness, disseminating these programs into community settings has been limited (8–10).

Community-wide and informational approaches to promoting physical activity behavior change can reach a large number of people with modest effort. Email-based intervention studies also show these strategies can be a cost-effective way of disseminating a physical activity intervention to a wide range of individuals. Furthermore, self-instructional behavior change programs are likely to reach more people than traditional face-to-face methods (11).

Effective dissemination of evidenced-based physical activity interventions require organizational commitment to ensure appropriate reach and implementation (8). One network that could facilitate walking promotion is the Cooperative Extension System (10, 12). The Cooperative Extension System, herein referred to as Extension, operates through the Land-Grant System across the U.S. Originally created to apply research and provide education in agriculture; Extension has grown dramatically since it began over 100 years ago. Since its inception, Extension has expanded its focus to include other areas of education and outreach such as nutrition, youth development, and health, with the latter being the fastest growing sector of needs that Extension is called upon to help address. Extension draws upon expertise and research from the land-grant institutions to support and provide hands-on, community-based solutions and programs to address a variety of needs across rural and urban communities. The purpose of this study was to evaluate the statewide dissemination of *Get WalkIN'*, an email-based walking promotion program delivered through Extension.

## Methods

### Theoretical Framework

A complete description of the guiding theoretical framework for *Get WalkIN'* has been previously published (13). In brief, *Get WalkIN'* is based on an interpersonal health behavior theory, social cognitive theory (14). Social cognitive theory emphasizes the dynamic interaction between personal factors, the health behavior, and the social environment (14). Self-efficacy is considered the main construct in social cognitive theory, which refers to an individual's confidence in his/her ability to perform a behavior (15). Self-efficacy also encompasses the ability to overcome barriers to the behavior and exertion of self-control of the behavior through the use of self-regulation and goal setting. Self-efficacy directly influences physical activity behavior and can act as a mediator by indirectly influencing behavior through other theoretical constructs such as social support (16).

### Intervention and Procedures

In 2016, *Get WalkIN'* was pilot tested for use by Extension before statewide implementation. This process of intervention development has been described in detail elsewhere (13). During program evaluation, Extension Educators who were involved in the pilot program agreed that *Get WalkIN'* was easy to implement and stated they planned to implement *Get WalkIN'* again in their community (17).

To begin statewide dissemination and assist with intervention fidelity, an intranet site was established and made accessible to all Extension Educators. This site provided necessary materials for participant recruitment and program implementation. Prior to intervention implementation, all Extension Educators watched a program training video. This training video included an overview of the theoretical framework for the program as well as the history of program development and testing. The video also guided Educators through the intranet site, demonstrated how to send the program emails, and reviewed the process for program evaluation.

Materials available on the intranet site included marketing and recruitment tools (i.e., flyers, newsletters, newspaper articles, social media messages) and additional resources and handouts that could be shared with participants. Educators were also provided with a program toolkit to facilitate effective program delivery in their respective target communities. *Get WalkIN'* includes 16 pre-developed email messages that are sent by county-based Extension Educators over the course of 12 weeks. These email messages are sent two times a week for the first 4 weeks and then once a week for the next 8 weeks. Messages target social cognitive theory constructs of self-efficacy, goal-setting, self-monitoring, and social support. Email messages also include prompts for participants to ask questions, share feedback, or send in photos of their walks. Participants could choose how involved or responsive they would like to be with the Extension Educator. Extension Educators were asked to not alter the pre-developed content of the program emails, but were encouraged to add any supplementary county-specific content that could benefit their participants, such as details about local opportunities and events to foster walking.

### Sample and Recruitment

In the fall of 2017, *Get WalkIN'* was made available to all Extension Educators across the state of Indiana. In addition to the use of recruitment materials available on the intranet site, Extension Educators were also encouraged to recruit using current listservs and during existing Extension programming events. Being an adult, age 18 or older, was the only inclusion criteria for program participation.

### Design

We conducted a preintervention-postintervention community-based trial to determine the effectiveness of statewide dissemination of *Get WalkIN'*. As this intervention has previously demonstrated effectiveness in a small randomized controlled design (13), a comparison group was not utilized in this study. To assess the dissemination process we used the RE-AIM framework (reach, effectiveness, adoption, implementation, and maintenance) which provides a comprehensive approach for evaluating the potential public health impact of an intervention (18). The project was approved by the Purdue University Institutional Review Board.

### Measures

Participants were emailed a survey via Qualtrics^®^ prior to the start of the program (baseline), immediately post-intervention (at week 12), and at 3 months post-intervention (maintenance). The Qualtrics^®^ survey was compatible for both computer and smart phone access. Participant characteristics of age, gender, marital status, household income, race, ethnicity, education, height, and weight were assessed at baseline only. Since this was a community-based program, completion of baseline and post-program surveys were not required to receive program emails.

#### Reach

Reach of the intervention was calculated in two ways. First, county-level reach was calculated as a percent using the number of participants who enrolled in the program divided by the total number of adults in each participating county who reported no leisure-time physical activity (19). Second, to gain a perspective of state-wide reach, we calculated a percent using the number of participants enrolled in the program divided by the total number of Indiana adults who reported no leisure-time physical activity as reported by the 2019 county health rankings state summary (19).

#### Efficacy

Efficacy of the intervention was assessed by examining change in self-reported physical activity. Secondary measures were changes in theoretical constructs. The International Physical Activity Questionnaire Short Form (IPAQ-SF) was used to assess physical activity during a typical 7-day period (20). This self-report tool asks participants to report the average number of days they participate in walking, moderate, and vigorous activity in a typical week and the average duration in minutes per activity episode. To understand the impact of this program on overall physical activity levels, a weekly metabolic equivalent (MET) score was calculated according to IPAQ-SF scoring guidelines. The following values were used for scoring: walking × 3.3 METs, moderate physical activity × 4.0, and vigorous physical activity × 8.0. The psychometric properties of the IPAQ-SF have been extensively tested and are shown to provide repeatable data (ρ ~ 0.80) with acceptable criterion validity (ρ ~ 0.30) (20).

Self-efficacy and social support were measured using existing valid and reliable tools, modified specifically to walking behaviors (21, 22). Walking self-efficacy was assessed with two Likert-scale subscales: making time (five items) and resisting relapse (four items) (22). Previous psychometric testing has shown these subscales to be significantly correlated with reported exercise, supporting criteria validity, and demonstrated reliability (α= 0.83–0.85) (22).

Social support for walking was assessed with seven Likert-scale items about the perceived social interactions and activities to support walking received from both family and friends (21). Scores were averaged across all items in each subscale. Previous psychometric testing has shown these subscales to be significantly correlated with reported exercise, supporting criteria validity, and demonstrated reliability (α= 0.84–0.91) (21).

#### Adoption

Adoption rate was calculated as a percent using the number of Extension Educators who implemented the program within 2 years of program launch divided by the total number of Extension Educators in Indiana.

#### Implementation

Implementation was assessed by tracking program start and end dates as well as the date for the maintenance email message to be sent. In addition, all Extension Educators were asked to submit a protocol form via Qualtrics^®^. This form asked Educators to acknowledge that the program was intended only for adults, attest they have watched the online training video, and that they agree to deliver the *Get WalkIN'* program in its entirety.

#### Maintenance

Maintenance of the program was calculated as the number of Extension Educators who ran the program a second time within the 2-year period divided by the number of Educators who ran the program at least once.

### Analysis

Participant characteristics and outcome measures were summarized with descriptive statistics. Means and standard deviations were calculated for continuous variables and frequencies and percentages were calculated for categorical variables. Chi-square and *t*-tests were used to assess differences between baseline and both post-intervention assessments (immediately post-intervention and 3 months post-intervention). Outliers in the self-reported walking, moderate, and vigorous activity were identified as scores exceeding z = |3.29| SD above the mean. These extreme values were winsorized to the next highest score of the range of the distributions (23). To examine the relationship between changes in self-efficacy and social support with changes in both weekly minutes of walking and weekly MET scores pre-post program, simple linear regression was conducted. Multivariate analysis was not appropriate due to multicollinearity between self-efficacy and social support. Data were analyzed using SAS 9.4 with statistical significance set at *p* < 0.05.

## Findings

### Reach and Sample

**Figure 1:**
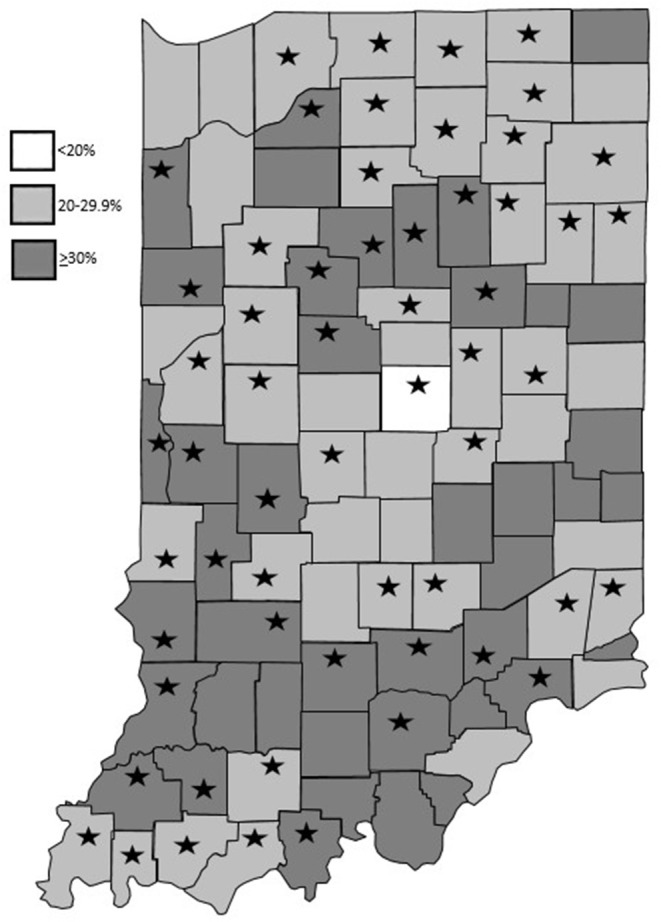
Program locations and percentage of residents who do not participate in any leisure time physical activity based on County Health Rankings data (19).

County-level reach was 0.08% and state-level reach was 0.04%. County Health Rankings estimates that 25% of Indiana adults (1,289,219 adults) participate in no leisure time physical activity (19). Between August 2017 to May 2019, 36 Extension Educators recruited 560 participants across 58 counties. Figure 1 depicts the locations of the participating counties throughout the state as well as the percentage of residents who did not participate in any leisure time physical activity according to county health rankings (19).

These participants were on average female (89%), middle aged (53 ± 23 years), and non-Hispanic white (95%). All participants completed high school or obtained a GED and 45.5% obtained at least a 4-year college degree (see Table 1). Twenty-six percent of respondents indicated a household income of less than $50,000 per year and 25% reported a household income of greater than $90,000 per year. In comparison, 79.2% of Indiana adults are non-Hispanic white, 88.3% are high school graduates, 25% have bachelor's degrees, and the median household income is $52,182 (24).

**Table 1:** Baseline sociodemographic and physical activity characteristics of participants (*n* = 560).

Age (mean years ± SD)	52.8 ± 23.4	
BMI (mean ± SD)	30.9 ± 7.5	
	n^*^	%
Gender		
Male	50	8.9
Female	498	88.6
Race		
White	531	94.5
Other	18	3.2
Marital status		
Married/Living as married	422	75.1
Single	53	9.4
Divorced/Separated	39	6.9
Widowed	28	5.0
Income		
< $50,000	144	25.6
$50,000–89,999	179	32.0
$90,000+	140	24.9
Education Level		
High school/GED	86	15.3
Some college/Technical school	117	20.8
2- or 4-year college degree	226	40.2
Masters or Professional degree	118	21.0
Doctoral degree	4	0.7

* *Columns do not equal full sample size due to missing data*.

### Efficacy

At baseline, participants reported walking an average of 115.2 ± 97.1 min per week. This significantly increased post-program to 187.2 ± 135.5 min per week. This increase was maintained 3 months post-program with participants reporting an average of 168.8 ± 108.5 min of walking per week (*p* < 0.05). Participants also reported significantly increased moderate and vigorous physical activity. At baseline, participants reported an average of 67.0 ± 64.3 min of moderate intensity activity per week. This significantly increased post-program to 107.6 ± 85.9 min per week and was maintained at the 3-month post-program assessment with participants reporting an average of 115.2 ± 88.7 min of moderate physical activity per week (*p* < 0.05). Vigorous physical activity also increased. At baseline, participants reported an average of 60.1 ± 69.2 min per week of vigorous physical activity. This significantly increased post-program to 102.7 ± 94.1 min per week, but decreased to 81.6 ± 77.4 min per week at the maintenance assessment (*p* < 0.05). Participants' average weekly MET score also significantly increased post-program from 1126.7 ± 909.0 to 1863.8 ± 1236.9 (*p* < 0.05). This increase was maintained during the 3-month post-program assessment (1676.15 ± 1039.12; *p* < 0.05).

Both self-efficacy subscales significantly increased from baseline to post-program and this increase was maintained during the maintenance assessment (see Table 2). On average, there was a significant increase in the resisting relapse subscale of self-efficacy from baseline to post-intervention (2.98 ± 0.75 vs. 3.30 ± 0.82; *p* < 0.05) which was maintained 3 months post-program (3.23 ± 0.89). Similar outcomes were seen in the making time subscale of self-efficacy which increased from 3.10 ± 0.75 to 3.35 ± 0.82 (*p* < 0.05) immediately post-program. This increase was maintained 3 months post-program (3.35 ± 0.78; *p* < 0.05). Participants also reported a significant increase in social support for physical activity immediately post-program (2.46 ± 0.98 vs. 2.80 ± 1.11; *p* < 0.05). However, reports of social support for physical activity decreased during the maintenance assessment which were not significantly different from baseline measures (2.68 ± 1.00; *p* > 0.05).

**Table 2:** Means and standard deviations of physical activity and theoretical constructs at baseline and post-intervention time points.

	**Baseline (*n* = 560)**	**Post-intervention (*n* = 205)**	**3 month maintenance (*n* = 117)**
	**Mean ± SD**	**Mean ± SD**	**Mean ± SD**
Self-efficacy			
Making Time	3.10 ± 0.75	3.35 ± 0.82^*^	3.35 ± 0.78^¥^
Relapse	2.98 ± 0.79	3.30 ± 0.83^*^	3.23 ± 0.89^¥^
Social Support	2.46 ± 0.98	2.81 ± 1.11^*^	2.68 ± 1.00
Weekly physical activity			
Vigorous	60.07 ± 69. 22	102.66 ± 94.11^*^	81.61 ± 77.39^¥^
Moderate	67.05 ± 64.30	107.6 ± 85.88^*^	115.17 ± 88.67^¥^
Walking	115.22 ± 97.13	187.18 ± 135.45^*^	168.75 ± 108.47^¥^
MET	1,126.65 ± 908.96	1,863.78 ± 1236.87^*^	1,676.15 ± 1,039.12^¥^

*MET, metabolic equivalent. Significant mark of difference between time points: Difference from previous time*:

* *p < 0.05; Difference from baseline*:

¥ *p < 0.05*.

When examining the relationship between changes in theoretical constructs with changes in weekly minutes of walking, increases in the making time self-efficacy subscale were significantly associated with increased walking immediately post-program (β = 11.29 ± 16.1; *p* < 0.05). No significant associations between resisting relapse and social support with weekly minutes of walking were found at either time points. A similar pattern was seen when examining the relationship between changes in weekly METs with changes in the theoretical constructs. Increases in the making time self-efficacy subscale were significantly associated with an increased MET score immediately post-program (β = 618.2 ± 95.6; *p* < 0.01) and during the maintenance assessment (β = 644.6 ± 119.1; *p* < 0.01). No significant associations between resisting relapse and social support with weekly MET scores were found at either time points.

### Adoption

The adoption rate for this program was 42.4%, 36 out of 85 Extension Educators implemented *Get WalkIN'* at least once during the 2-year dissemination period.

### Implementation

Before program implementation occurred at the county-level, each Extension Educator completed a protocol form, agreeing to deliver *Get WalkIN'* in its entirety, including all 16 email messages and the 3-month maintenance email. In addition, the lead researcher monitored program start and end dates for each Educator and individually contacted Educators to track response rates.

### Maintenance

The maintenance rate for this program was 30.6%, 11 out of 36 Extension Educators who implemented *Get WalkIN'* during the dissemination period conducted the program at least twice.

## Discussion

The purpose of this study was to evaluate the statewide dissemination of the *Get WalkIN'* program using the RE-AIM framework. The low reach of this program is likely due to the broad definitions of reach used. Kessler et al. (25) define reach more narrowly as the number of participants who enroll in a program divided by the number of participants who received information about the program. However, the Extension Educators used reactive recruiting for this program (i.e., flyers, emails, newsletters, social media). Therefore, it was not possible to accurately assess how many potential participants saw information about *Get WalkIN'*. This led to the use of a broad potential audience and hence, lower rates of reach.

Findings from this dissemination study suggest that *Get WalkIN'* is effective in increasing physical activity among participants. After the program, participating Indiana adults increased their walking and overall physical activity behavior and maintained this behavior change over the course of the 3-month post-program maintenance phase. Furthermore, the increase in weekly minutes of walking was not at the sacrifice of other forms of physical activity as the reported minutes of moderate and vigorous intensity activity remained stable or also increased.

Results are consistent with social cognitive theory as findings suggest that changes in theoretical constructs of self-efficacy and social support could at least partially contribute to the increases seen in walking and overall physical activity behavior. Participants reported significant increases in both self-efficacy and social support immediately after the program. Increases in self-efficacy were also maintained 3 months after the program during the maintenance assessment. Furthermore, the regression analysis indicates that the increases seen in the making time subscale of self-efficacy were significantly associated with increases in both weekly minutes of walking and the weekly MET score.

The adoption rate among Extension Educators was high. This suggests that the flexibility of an email-based program is attractive for Educators to deliver. However, the implementation of *Get WalkIN'* was not rigorously tracked. While each Extension Educator who implemented *Get WalkIN'* underwent online training and agreed to implement the program as intended, follow-up data on program fidelity is limited. We are unable to report the percentage of Educators who delivered the program according to the delineated timeline. The use of focus groups to obtain qualitative data from Extension Educators could provide additional insight into program implementation. Having Educators self-report on the implementation process through the use of a check-list would improve on the data available to assess the implementation process. Additionally, if funds allowed, site visits could be made to county Extension offices to more rigorously evaluate program implementation.

Maintenance, defined as the percentage of Educators who offered *Get WalkIN'* more than once, was moderate. Several factors could have influenced the decision to implement *Get WalkIN'* more than once. First, given the impact of seasonality on walking (26), some Educators may have chosen to offer the program during fair weather months only. Second, it is possible that there was staff turnover in the county Extension offices that impacted repeat delivery of the program.

There are limitations that should be considered in light of study findings. First, as this was a community-based health promotion program, completing pre and post surveys was not required for program participation. This likely attributed to participant attrition. At baseline, 560 participants completed baseline surveys, but only 205 participants completed the first follow-up survey immediately post-program, and 117 participants completed the 3-month maintenance survey. It has been recognized that the evaluation of community-based behavior change programs often involves practical constraints on obtaining follow-up data (8, 12). However, it remains important to note that reporting and non-response bias may be seen in the results as previous studies have shown that participants who are early responders to follow-up surveys do not tend to be representative of the entire population of participants (12, 27). Furthermore, the demographic characteristics of participants was assessed only at baseline so comparison between responders and non-responders is not possible. To address issues with attrition in future evaluation efforts, it may be necessary to stress the importance of evaluation and follow-up data with program participants. It has been suggested that fostering a sense of empowerment in program participants can facilitate more complete evaluation data (28). While it will add cost to the program, the provision of incentives or prizes for program completion might increase participant retention rates.

Despite recruitment materials displaying images of diverse populations, *Get WalkIN'* attracted participants that were predominantly white (95%) females (89%), which affects the external validity and public health relevance of the findings. However, in our state, this population is a large proportion of those served through Extension programming. It is also important to note that many of the participants who enrolled in this walking program were moderately physically active prior to the start of the program. At baseline, participants reported an average of 115 min of walking per week. This suggests that *Get WalkIN'* generally attracted participants who were more physically active, which limits the generalizability to a broader more inactive audience. To further examine the effectiveness of this program in more diverse populations, future implementations of this program will target more vulnerable audiences such as those residing in rural areas, those who have lower incomes and/or are considered racial or ethnic minorities. In addition, assessment of maintenance of behavior change should extend beyond 3 months.

As with many community-based physical activity programs, this study utilized self-report measures to assess physical activity behavior. These self-report measures are known to be prone to biases such as self-report, social desirability, testing, and recall bias (29). These biases may be reflective in the overall higher weekly physical activity minutes reported at baseline. However, the self-report measures used in this study have been extensively tested and are considered to be reliable and valid measures (20). Objective measures of physical activity using accelerometers or consumer-based activity monitors could be considered in future studies; however, objective measures are not always feasible or appropriate in population level studies (30, 31).

While email-based health promotion studies can demonstrate effectiveness, technology is rapidly advancing and other distance-based mechanisms may be more appropriate. For example, in the era of smartphones and immediate response, text messaging could prove to be just as or an even more effective means of program implementation. However, the work for Educators or other program implementation staff may be more burdensome to send text messages than utilizing email. Furthermore, sending text messages could introduce an additional cost to both Extension and participants.

There are also strengths associated with this study that enhance findings. First, a partnership with an established county-based network of Educators through Cooperative Extension, provided the ability to reach large segments of the population. Successful intervention dissemination requires strong community partnerships such as Cooperative Extension (9). Second, *Get WalkIN'* email messages were created focusing on social cognitive theory constructs, which are well-studied in physical activity behavior change research (32). Past community-based health promotion programs (28), including physical activity interventions (32, 33), have not consistently utilized health behavior theories in program development and evaluation. Among participants who completed baseline and follow-up measures, *Get WalkIN'* significantly increased and maintained increases in self-efficacy, which is a key factor in increasing and maintaining behavior change. Further, *Get WalkIN'* was systematically pilot tested before statewide launch which likely facilitated the effectiveness of this program (13, 17). Additionally, email delivery of this program is easily transferable across settings and populations. Finally, the implementation costs for this program are low and the community buy-in for implementing this intervention is high, as evidenced by the high number of county-based Extension Educators electing to offer the program.

*Get WalkIN'* is an example of a community-based program derived from research, grounded in data supporting evidence of effectiveness, and making an impact through delivery by Extension Educators. This program also addresses the ability to be more equitable in providing health education programs, bringing more health and wellness opportunities to rural communities and residents through its email-based delivery mechanism, and its use of county-based Extension Educators. Community physical activity programs such as *Get WalkIN'* have the potential to increase physical activity levels and decrease sedentary behaviors. With continued evaluation and updates, these types of programs can also create a sense of community and synergy among participants, which in turn, could contribute to high program engagement. Further, community-based programs can also facilitate community linkages as many community physical activity programs are provided by community-based organizations such as health clinics, non-profit organizations, and hospitals. Cooperative Extension is a natural partner for such work given the strong investment in community health and existing linkages with residents and existing community-based programs.

## Data Availability Statement

The datasets for this article are not publicly available because of institutional review board restrictions. Requests to access the datasets should be directed to erichards@purdue.edu.

## Ethics Statement

The studies involving human participants were reviewed and approved by Purdue University Human Research Protection Program. Written informed consent for participation was not required for this study in accordance with the national legislation and the institutional requirements.

## Author Contributions

ER and SW conceptualized the study and contributed to the collection, analysis, and interpretation of data as well as manuscript development. AF contributed to manuscript preparation. All authors read and approved the final manuscript.

### Conflict of Interest

The authors declare that the research was conducted in the absence of any commercial or financial relationships that could be construed as a potential conflict of interest.
